# Supplementary material for: Hybrid Models and Biological Model Reduction with PyDSTool
Source: PLoS Comput Biol. 2012 Aug 9;8(8):e1002628. doi: 10.1371/journal.pcbi.1002628 (PMC3415397; doi:10.1371/journal.pcbi.1002628)
Supplement: Text S4 — Complete source code for the PyDSTool package (version 0.88.120504). Includes API documentation and help files linking to web pages. This file is identical to the current public release on Sourceforge.net. (ZIP) [file pcbi.1002628.s004.zip › PyDSTool/html/PyDSTool.Toolbox.ModelHelper-pysrc.html]

xml version="1.0" encoding="ascii"?


PyDSTool.Toolbox.ModelHelper


| Home | Trees | Indices | Help | PyDSTool |
| --- | --- | --- | --- | --- |

Package PyDSTool ::
Package Toolbox ::
Module ModelHelper

[hide private]

[frames] | no frames]

# Source Code for Module PyDSTool.Toolbox.ModelHelper

```
  1  """A set of functions to help build ODE models with complete 
  2    sets of standard events, etc. based on function specifications 
  3    provided in a format similar to that for sloppyModels. 
  4   
  5    Eric Sherwood. 
  6   
  7   
  8    2008: This is superceded by GDescriptor class. See PyDSTool/tests/ 
  9  """ 
 10  from PyDSTool import * 
 11  from copy import copy 
 12  from time import clock 
 13   


14 -class SpiffyODEModel(LeafComponent):


15      pass

 16   
 17  # Set of all compatible generators 
 18  allODEgens = findGenSubClasses('ODEsystem') 
 19   


20 -def makeSpiffyODEModel(**kw):


21   
 22      if 'modelName' in kw: 
 23          modelName = kw['modelName'] 
 24      else: 
 25          raise 'No modelName specified' 
 26   
 27      if 'modelDict' in kw: 
 28          modelDict = kw['modelDict'] 
 29      else: 
 30          raise 'No modelDict specified' 
 31   
 32      if 'targetGen' in kw: 
 33          targetGen = kw['targetGen'] 
 34      else: 
 35          # Default to Dopri_ODEsystem 
 36          targetGen = 'Dopri_ODEsystem' 
 37   
 38      if 'globalRefs' in kw: 
 39          globalRefs = kw['globalRefs'] 
 40      else: 
 41          globalRefs = [] 
 42          kw['globalRefs'] = globalRefs 
 43   
 44      if 'algParams' in kw: 
 45          algParams = kw['algParams'] 
 46      else: 
 47          algParams = {'init_step':0.05, 'max_pts':5000000, 'atol': 1e-12, 'rtol': 1e-12} 
 48          kw['algParams'] = algParams 
 49   
 50      if 'eventDefaults' in kw: 
 51          eventDefaults = kw['eventDefaults'] 
 52      else: 
 53          eventDefaults = { 
 54              'starttime': 0, 
 55              'term': False} 
 56          kw['eventDefaults'] = eventDefaults 
 57   
 58      if 'eventTol' in kw: 
 59          eventTol = kw['eventTol'] 
 60      else: 
 61          eventTol = 1e-9 
 62          kw['eventTol'] = eventTol 
 63   
 64      if 'indepVar' in kw: 
 65          indepVar = kw['indepVar'] 
 66      else: 
 67          indepVar = ('t', [0, 100000]) 
 68          kw['indepVar'] = indepVar 
 69   
 70      if 'withEvents' in kw: 
 71          withEvents = kw['withEvents'] 
 72      else: 
 73          withEvents = True 
 74          kw['withEvents'] = withEvents 
 75   
 76      if 'withJac' in kw: 
 77          withJac = kw['withJac'] 
 78      else: 
 79          withJac = False 
 80          kw['withJac'] = withJac 
 81   
 82      if 'withJacP' in kw: 
 83          withJacP = kw['withJacP'] 
 84      else: 
 85          withJacP = False 
 86          kw['withJacP'] = withJacP 
 87   
 88      if 'silent' in kw: 
 89          silent = kw['silent'] 
 90      else: 
 91          silent = True 
 92          kw['silent'] = silent 
 93   
 94      if 'buildModel' in kw: 
 95          buildModel = kw['buildModel'] 
 96      else: 
 97          buildModel = False 
 98          kw['buildModel'] = buildModel 
 99   
100      if 'buildCont' in kw: 
101          buildCont = kw['buildCont'] 
102      else: 
103          buildCont = False 
104          kw['buildCont'] = buildCont 
105   
106   
107   
108      makeJac = withJac 
109      makeJacP = withJacP 
110   
111      if targetGen not in allODEgens: 
112          # Print a list of the valid targets 
113          print 'Valid target ODE solvers: ' + ", ".join(allODEgens) 
114          raise ValueError('Invalid target ODE solver') 
115   
116      # Empty model spec to be populated with the information in modelDict 
117      theModelSpec = SpiffyODEModel(modelName, compatibleGens=allODEgens) 
118   
119      if not silent: 
120          print "Building model: '%s'"%modelName 
121   
122      # Build the ODE RHS, save list of ODEs 
123      varnames = [] 
124      if 'odes' in modelDict.keys(): 
125          for odeName, expr in modelDict['odes'].iteritems(): 
126              theModelSpec.add(Var(expr, odeName, specType='RHSfuncSpec')) 
127              varnames.append(odeName) 
128              if not silent: 
129                  print 'Added ODE: ', odeName 
130   
131      # Build the aux variables, save list of them 
132      auxvarnames = [] 
133      if 'auxs' in modelDict.keys(): 
134          for auxName, expr in modelDict['auxs'].iteritems(): 
135              theModelSpec.add(Var(expr, auxName, specType='ExpFuncSpec')) 
136              auxvarnames.append(auxName) 
137              if not silent: 
138                  print 'Added aux variable: ', auxName 
139   
140      # Build the list of RHS specific parameters, save list of them 
141      parnames = [] 
142      if 'params' in modelDict.keys(): 
143          for parName, val in modelDict['params'].iteritems(): 
144              theModelSpec.add(Par(str(val), parName)) 
145              parnames.append(parName) 
146              if not silent: 
147                  print 'Added parameter: ', parName, "=", val 
148   
149      # Build the inputs, save list of them 
150      inputnames = [] 
151      inputdict = {} 
152   
153      if 'inputs' in modelDict.keys(): 
154          for inputName, val in modelDict['inputs'].iteritems(): 
155              theModelSpec.add(Input(str(inputName))) 
156              inputnames.append(inputName) 
157              #if not silent: 
158              #    print val.variables.keys() 
159              inputdict[inputName] = val.variables[inputName] 
160              if not silent: 
161                  print 'Added input: ', inputName, "=", val 
162   
163      # Build the dict of extra functions, save list of them 
164      auxfndict = {} 
165      if 'functions' in modelDict.keys(): 
166          for funSig, expr in modelDict['functions'].iteritems(): 
167              # check syntax; this could be taken care of by Lex-built 
168              # syntax checking function 
169              assert ')' == funSig[-1] 
170              assert '(' in funSig 
171              # Not sure what this replacement is doing? 
172              major = funSig.replace(')', '').replace(' ', '').split('(') 
173              args = major[1].split(',') 
174              name = major[0] 
175   
176              theModelSpec.add(Fun(expr, args, name)) 
177              auxfndict[name] = (args, expr) 
178              # Jacobians are special and might otherwise be handled separately 
179              if name == 'Jacobian': 
180                  makeJac = False 
181              if name == 'Jacobian_pars': 
182                  makeJacP = False 
183              if not silent: 
184                  print 'Added function: ', name, " of arguments: ", args 
185   
186   
187      # Make symbolic jacobian if requested 
188      if makeJac: 
189          if 'Jacobian' not in modelDict.keys(): 
190              varnames.sort() 
191              dotlist = [modelDict['odes'][x] for x in varnames] 
192              F = Fun(dotlist, varnames, 'F') 
193              Jac = Fun(Diff(F, varnames), ['t'] + varnames, 'Jacobian') 
194              theModelSpec.add(Jac) 
195              if not silent: 
196                  print "Added jacobian" 
197   
198      if makeJacP: 
199          if 'Jacobian_pars' not in modelDict.keys(): 
200              varnames.sort() 
201              parnames.sort() 
202              dotlist = [modelDict['odes'][x] for x in varnames] 
203              G = Fun(dotlist, varnames + parnames, 'G') 
204              JacP = Fun(Diff(G, varnames + parnames), ['t'] + varnames + parnames, 'Jacobian_pars') 
205              theModelSpec.add(JacP) 
206              if not silent: 
207                  print "Added jacobian w.r.t parameters" 
208   
209   
210      # Construct the model 
211      targetLang = theGenSpecHelper(targetGen).lang 
212      # single-generator model so give both same name 
213      genName = modelName 
214   
215      theModel = ModelConstructor(modelName, 
216                                  generatorspecs={genName: {'modelspec': theModelSpec, 
217                                                            'target': targetGen, 
218                                                            'algparams': algParams 
219                                                            }}, 
220                                  indepvar=indepVar, 
221                                  inputs=inputdict, 
222                                  eventtol=eventTol, withStdEvts={genName: withEvents}) 
223   
224      return theModel

225
```

  


| Home | Trees | Indices | Help | PyDSTool |
| --- | --- | --- | --- | --- |

|  |  |
| --- | --- |
| Generated by Epydoc 3.0.1 on Fri May 4 15:24:16 2012 | http://epydoc.sourceforge.net |
